# Trends and disparities in ischemic stroke mortality and location of death in the United States: A comprehensive analysis from 1999–2020

**DOI:** 10.1371/journal.pone.0319867

**Published:** 2025-04-09

**Authors:** Jason K. Lim, Jenlu Pagnotta, Richard Lee, Do H. Lim, Jeffrey M. Breton, Zachary A. Abecassis, Raymond M. Meyer, Jeffrey C. Mai, Michael R. Levitt

**Affiliations:** 1 Department of Neurological Surgery, Georgetown University, District of Columbia, Washington, United States of America; 2 Department of Bioengineering, University of Pennsylvania, Philadelphia, Pennsylvania, United States of America; 3 Department of Neurological Surgery, University of Washington, Seattle, Washington, United States of America; 4 Departments of Neurological Surgery, Radiology, Mechanical Engineering, Neurology, and Stroke & Applied Neuroscience Center, University of Washington, Seattle, Washington, United States of America; Chinese Academy of Medical Sciences and Peking Union Medical College, CHINA

## Abstract

**Background:**

Stroke remains the fifth leading cause of mortality in the United States, with significant geographical and racial disparities in outcomes. Understanding trends in location of death for ischemic stroke patients is crucial for improving end-of-life care and addressing healthcare inequities.

**Methods & Findings:**

This retrospective study used Centers for Disease Control and Prevention’s Wide-ranging Online Data for Epidemiologic Research (CDC WONDER) data to examine ischemic stroke mortality, stratified by urbanization level and race. Age-adjusted mortality rates were calculated using the 2000 US standard population. Age-adjusted ischemic stroke mortality rates increased across all urbanization levels since 2009, with the most pronounced rises in non-metropolitan areas. An increasing proportion of deaths occurred at home, shifting from inpatient medical facilities. Significant disparities were observed in access to specialized end-of-life stroke care, particularly for racial minorities and rural residents. Black/African American individuals and those in rural settings were more likely to die in less specialized environments due to healthcare access barriers.

**Conclusions:**

The findings highlight a critical shift in the patterns of mortality and end-of-life care preferences among ischemic stroke patients over the past two decades. These findings highlight significant shifts in the patterns of mortality and location of death among ischemic stroke patients over the past two decades, with notable differences across urbanization levels and racial groups. The increasing proportion of home deaths and persistent disparities in location of death suggest a need for further research to understand the underlying factors driving these trends and their implications for end-of-life care quality and access.

## Introduction

In the United States, stroke constitutes the fifth leading cause of mortality, resulting in around 150,000 deaths in 2020 [1]. Recent analyses show indications of increased incidence and mortality since 2010 despite therapeutic advancements [2,3]. Furthermore, stroke remains a significant contributor to long-term disability and diminished quality of life, posing serious strains on healthcare systems [4].

Research has documented geographical disparities in stroke care access and outcomes [5]. Ischemic stroke carries a mortality rate of 10% within 30 days and up to 40% within one year. Survival is heavily dependent on time-sensitive treatment [6]. However, patients living in rural areas face barriers in reaching the specialized facilities that can provide timely and advanced care [1], and rural hospitals frequently lack dedicated resources to treat complex stroke cases or rehabilitate patients [7]. In addition, socioeconomic status, higher rates of comorbidities such as obesity and smoking, and demographic characteristics further complicate stroke care for racial minorities [6,8]. Consequently, studies consistently show elevated stroke mortality among rural residents even after adjusting for clinical and hospital factors [3,9,10]. These indications of worse outcomes underscore the importance of optimizing policies, infrastructure, and care connectivity for vulnerable stroke populations removed from urban stroke centers.

Location of death represents a surrogate to evaluate healthcare access, quality, and evolving end-of-life care preferences [11]. These data provide valuable insights into final care experiences for stroke patients. For rural patients already experiencing disadvantages in stroke care, location of death metrics may reflect avoidance or delay in securing advanced stroke treatments only offered in specialized facility settings. Unable to reach such facilities due to transfer barriers or limitations around thrombolytic administration timing, rural patients often face restricted treatment options [12]. By tracking the locations where stroke patients ultimately die, we can gauge disparities in healthcare access and quality, providing a more comprehensive understanding of the challenges in stroke care delivery across different populations and regions.

Previous research has explored trends in location of death for individuals with cerebrovascular disease in the United States. A study by Cross et al. using the Centers for Disease Control and Prevention’s Wide-ranging Online Data for Epidemiologic Research (CDC WONDER) database from 2003 to 2017 found increasing proportions of deaths occurring at home and in hospice facilities, with racial and ethnic disparities in these trends [13]. More recently, McCandless et al. analyzed stroke mortality trends among older adults from 1999 to 2020, revealing distinct patterns for different stroke types. Notably, they found that cerebral infarction mortality decreased from 1999 through 2014, followed by a rapid increase through 2017 before stabilizing [14]. Our study builds upon these findings by focusing specifically on ischemic stroke and examining the interplay between race, ethnicity, and urbanization level in end-of-life care over two decades.

This study aims to evaluate trends in the location of death among ischemic stroke decedents in the U.S. from 1999-2020, with a particular focus on disparities by urbanization level and race. To do so, we analyzed data from the CDC WONDER database, examining three main indicators: number of deaths, age-adjusted ischemic stroke mortality, and location of deaths. While our primary focus is on location of death, analyzing mortality rates and death counts provides essential context for interpreting changes in death location over time and across different groups. Stratifying by rural-urban status and race, we hypothesized that persistent disparities are present in access to facility-based stroke care at end-of-life, with higher proportions of home deaths among rural and minority residents. This comprehensive approach allows us to understand how these indicators are interconnected, providing a more nuanced view of ischemic stroke outcomes and disparities.

## Methods

### Data source and classification

This retrospective study utilized data from the CDC WONDER database to examine trends related to ischemic stroke mortality from 1999 to 2020 [15]. We specifically analyzed the CDC WONDER Underlying Cause-of-Death records, which comprise nationwide death certificate data [15]. The study was conducted in compliance with reporting guidelines and exempted from ethics board review given the publicly available, deidentified nature of the aggregate database.

### Case identification

We focused on U.S. residents of all ages whose underlying cause of death was classified as ischemic stroke based on the International Classification of Diseases, 10th Revision (ICD-10) codes I63.0-I63.9 recorded on death certificates [15].

### Variables and definitions

We categorized deceased individuals by sex, race, age group, and urbanization level. The CDC WONDER database has four races: American Indian/Alaska Native, Asian/Pacific Islander, Black/African American, and White. While the database includes Hispanic ethnicity data, we did not incorporate this category in our analysis for several reasons. First, the bridged-race methodology used by CDC WONDER assigns multiracial individuals to one of the four main race categories to ensure consistency across different data sources and years [16,17]. Second, using these broader race categories helps maintain statistical reliability, especially when analyzing data across multiple variables and geographic areas [16].

The 2013 Urban-Rural Urbanization scheme was established by the National Center for Health Statistics where every county is categorized into one of the six urbanization groups: Noncore, Micropolitan, Small Metro, Medium Metro, Large Fringe Metro, and Large Central Metro [17]. These urbanization groups were consolidated into two rural-urban tiers: Metropolitan (Small Metro, Medium Metro, Large Fringe Metro, and Large Central Metro) and Nonmetropolitan (Micropolitan and NonCore).

The location of death on the death certificates was largely classified into medical facility-based or non-medical facility-based. The medical facility base included the following subgroups: dead on arrival, inpatient, outpatient, or ER, and status unknown. The non-facility-based locations included the decedent’s home, hospice facility, nursing home/long-term care, other, and place of death unknown [17].

### Statistical analyses

We calculated crude and age-adjusted mortality rates across all population subgroups. Trends over time were evaluated by obtaining age-adjusted mortality rates per 100,000 people using the 2000 U.S. standard population, as defined by the CDC. Crude mortality rates are misleading because some regions and states have a relatively younger or older population. The CDC WONDER database indicates that age-adjusted rates are significant for comparing different subgroup populations and emphasizes that these rates should be considered relative indices instead of actual mortality risks [18].

In our analysis, we examined the interrelation between three key indicators: number of deaths, age-adjusted ischemic stroke mortality, and location of deaths. The number of deaths provided a baseline understanding of the scale of ischemic stroke mortality, while age-adjusted mortality rates allowed for fair comparisons across demographic groups and time periods. Location of deaths, our primary focus, offered insights into healthcare access, quality, and end-of-life care preferences. These indicators were analyzed collectively to provide a comprehensive picture of ischemic stroke outcomes and disparities.

## Results

### Mortality distribution by urbanization, race, sex, and age

From 1999 to 2020, 237,617 ischemic stroke deaths were recorded. Large Central Metro areas had the highest number of deaths (62,224; 26.13%) while Noncore (Nonmetro) had the lowest number of deaths (19,254; 8.09%). White individuals had the highest number of deaths (203,198; 85.51%), followed by Black/African American (28,540; 12.01%), Asian/Pacific Islander (5,638; 2.37%), and American Indian/Alaska Native individuals (241; 0.10%). Compared to the 2020 population estimates, White individuals were overrepresented in deaths by 8.12% points, while other races were underrepresented. Mortality was greater in women (138,768; 58.39%) than men (98,849; 41.60%). The oldest age group (≥85 years) had the highest number of deaths (98,325; 41.55%). Inpatient medical facilities were the most common place of death (106,348; 44.65%), followed by nursing homes/long-term care facilities (63,414; 26.63%) and death at home (34,691; 14.57%) (Table 1).

**Table 1:** Total mortality distribution categorized by urbanization, race, sex, and age 1999-2020.

Category	Subcategory	Deaths (Number)	Percent (%)
**Urbanization**	Large Central Metro	62,224	26.13%
	Large Fringe Metro	53,115	22.31%
	Medium Metro	53,508	22.47%
	Micropolitan (Nonmetro)	26,164	10.99%
	NonCore (Nonmetro)	19,254	8.09%
	Small Metro	23,864	10.02%
**Sex**	Women	138,768	58.40%
	Men	98,849	41.60%
**Age Groups**	< 1 year	10	0.00%
	1-4 years	0	0.00%
	5-14 years	0	0.00%
	15-24 years	32	0.01%
	25-34 years	652	0.28%
	35-44 years	2,913	1.23%
	45-54 years	8,453	3.57%
	55-64 years	19,221	8.12%
	65-74 years	36,237	15.13%
	75-84 years	70,799	29.92%
	85 + years	98,325	41.55%
**Place of Death**	Medical Facility - DOA	151	0.06%
	Medical Facility - Unknown	200	0.08%
	Unknown	245	0.10%
	Medical Facility - Outpatient	4,187	1.76%
	Other	9,063	3.81%
	Hospice facility	19,830	8.33%
	Decedent’s home	34,691	14.57%
	Nursing home/long term care	63,414	26.63%
	Medical Facility - Inpatient	106,348	44.66%
**Race**	American Indian or AK Native	241	0.10%
	Asian or Pacific Islander	5,638	2.37%
	Black or African American	28,540	12.01%
	White	203,198	85.51%

### Trends in age-adjusted death rates by race (1999-2020)

**Fig 1:**
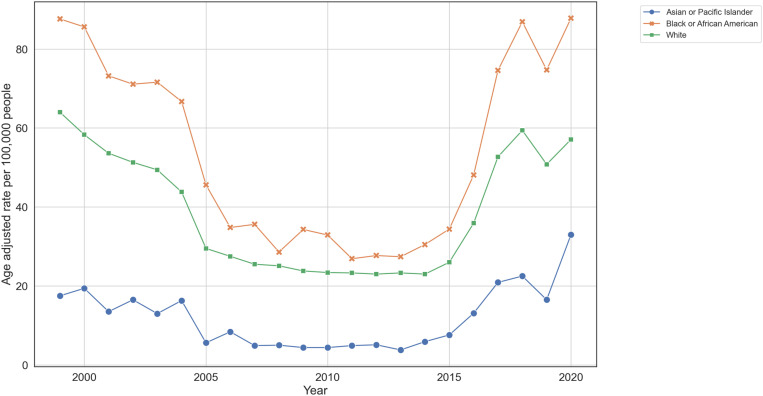
Age-adjusted death rates for ischemic strokes classified by race. 1999-2020. The graph shows the trends in age-adjusted death rates per 100,000 people for Asian/Pacific Islander (blue), Black/African American (orange), and White (green) populations. Data were obtained from the CDC WONDER database. Age-adjusted rates were calculated using the 2000 U.S. standard population.

Age-adjusted death rates from 1999 to 2020 demonstrated distinct trends among four racial groups over the 21-year period. However, our dataset does not include information on American Indian/Alaskan Natives because the CDC WONDER database suppresses rates when the annual death count is fewer than 20 individuals to maintain confidentiality.^18^ Among the three remaining groups, the Asian/Pacific Islander population had the lowest rate in 1999 (17.5 per 100,000) but nearly doubled by 2020 (33.0). Black/African American individuals had the highest rate in 1999 (87.6), which declined to 34.8 in 2006 but rose back to 87.8 by 2020. The White population showed a gradual decrease from 64.0 in 1999 to 57.1 in 2020. The Asian/Pacific Islander group had the least variability, while the Black/African American group saw the most decline followed by a significant rise back to initial levels (Fig 1).

### Place of death by urbanization level

**Fig 2:**
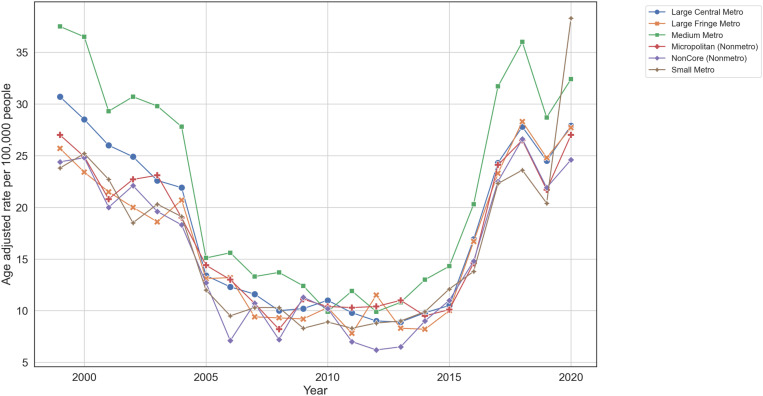
Age-adjusted death rates for ischemic strokes classified by urbanization level. 1999-2020. The graph displays the age-adjusted death rates per 100,000 people for different urbanization levels: Large Central Metro (blue), Large Fringe Metro (orange), Medium Metro (green), Small Metro (brown), Micropolitan (Nonmetro) (red), and NonCore (Nonmetro) (purple). Data were obtained from the CDC WONDER database. Age-adjusted rates were calculated using the 2000 U.S. standard population.

The distribution of places of death varied across urbanization levels, with inpatient medical facilities being the most common place of death across all categories, ranging from 41.65% in Large Fringe Metro areas to 52.03% in NonCore areas. The age-adjusted death rate was highest in Medium Metro areas for most years from 1999 to 2020, except for 2010, 2012, 2013, and 2020 (Fig 2). Deaths at home and in hospice facilities were more prevalent in urban areas, with Large Central Metro and Large Fringe Metro areas reporting higher percentages (15.93% and 15.28% at home, respectively; 7.33% and 9.78% in hospice facilities, respectively) compared to NonCore rural areas (11.68% at home, 4.58% in hospice facilities). Conversely, the percentage of deaths in nursing homes or long-term care facilities was higher in more rural settings, such as Micropolitan (30.25%) and NonCore areas (27.56%), compared to urban settings like Large Central Metros (24.82%) (Table 2).

**Table 2:** Location of death by urbanization level and race with number of deaths and percentage in parentheses. NaN =  missing or unidentified data due to less than 20 sample size.

	Decedent’s home	Hospice facility	Dead on Arrival	Medical Facility - Inpatient	Medical Facility - Outpatient or ER	Medical Facility - Status unknown	Nursing home/long term care	Other	Place of death unknown
**Urbanization Type**									
Large Central Metro	9,910 (15.93%)	4,560 (7.33%)	66 (0.11%)	28,433 (45.69%)	1,194 (1.92%)	98 (0.16%)	15,445 (24.82%)	2,489 (4.00%)	29 (0.05%)
Large Fringe Metro	8,116 (15.28%)	5,198 (9.78%)	25 (0.05%)	22,121 (41.65%)	933 (1.76%)	102 (0.19%)	14,248 (26.82%)	2,299 (4.32%)	85 (0.16%)
Medium Metro	7,805 (14.59%)	5,591 (10.45%)	50 (0.09%)	22,893 (42.78%)	928 (1.73%)	NaN	13,972 (26.12%)	2,245 (4.20%)	24 (0.04%)
Micropolitan (Nonmetro)	3,253 (12.43%)	1,477 (5.65%)	10 (0.04%)	12,284 (46.95%)	474 (1.81%)	NaN	7,915 (30.25%)	721 (2.76%)	30 (0.11%)
NonCore (Nonmetro)	2,248 (11.68%)	882 (4.58%)	NaN	10,017 (52.03%)	296 (1.54%)	NaN	5,306 (27.56%)	466 (2.42%)	39 (0.20%)
Small Metro	3,359 (14.08%)	2,134 (8.94%)	NaN	10,600 (44.42%)	362 (1.52%)	NaN	6,528 (27.35%)	843 (3.53%)	38 (0.16%)
**Race**									
American Indian/ Alaska Native	156 (14.87%)	63 (0.61%)	NaN	574 (54.72%)	27 (2.57%)	NaN	197 (18.78%)	32 (3.05%)	NaN
Asian/ Pacific Islander	1,062 (17.76%)	347 (5.80%)	10 (0.17%)	3,200 (53.51%)	132 (2.21%)	NaN	979 (16.37%)	250 (4.18%)	NaN
Black/ African American	4,140 (14.51%)	2,052 (7.19%)	106 (0.37%)	14,756 (51.70%)	927 (3.25%)	54 (0.19%)	5,601 (19.63%)	805 (2.82%)	99 (0.35%)
White	29,933 (14.44%)	17,434 (8.58%)	339 (0.17%)	87,818 (43.22%)	3,165 (1.56%)	209 (0.10%)	56,837 (27.87%)	7,985 (3.93%)	278 (0.14%)

### Place of death by race

**Fig 3:**
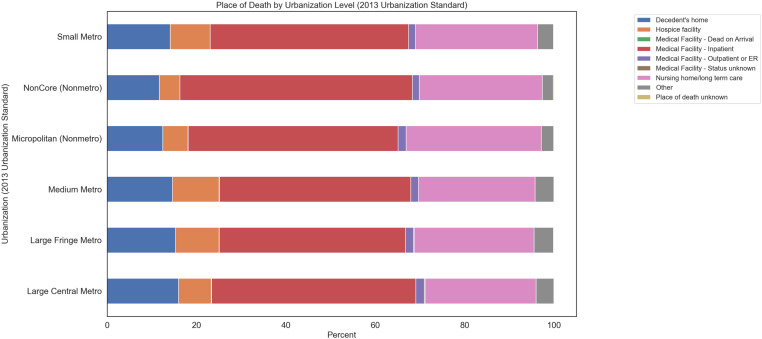
Ischemic stroke deaths classified by race and location of death. 1999-2020.

Ischemic stroke deaths classified by race and location of death showed that American Indian/Alaska Native individuals had the highest percentage of deaths in medical facilities with inpatient services at 54.72%. Asian/Pacific Islander individuals had the highest percentage of deaths at the decedent’s home at 17.76%. Black/African American individuals had the highest rate of death at medical facilities with death on arrival at 0.37% and the second highest death rate at hospice facilities and nursing home/long-term care at 7.19% and 19.63%, respectively. White individuals had the lowest death rate at medical facilities with inpatient or outpatient/ER services but the highest death rate at hospice facilities (8.58%) and nursing home/long-term care (87.87%) (Table 2, Fig 3).

The stacked bar graph shows the percentage of deaths occurring in different locations for American Indian/Alaska Native, Asian/Pacific Islander, Black/African American, and White populations. Data were obtained from the CDC WONDER database. Locations of death include Decedent’s home, Hospice facility, Dead on Arrival, Medical Facility - Inpatient, Medical Facility - Outpatient or ER, Medical Facility - Status unknown, Nursing home/long term care, Other, and Place of death unknown.

### Trends in deaths by place of death

**Fig 4:**
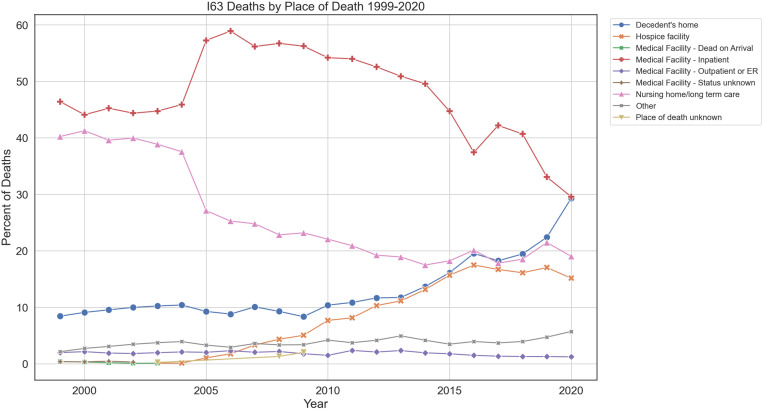
Percent of deaths for ischemic strokes classified by location of death, by year. 1999-2020. The graph displays the percentage of deaths occurring in different locations over time. Data were obtained from the CDC WONDER database. Locations of death include Decedent’s home, Hospice facility, Medical Facility - Dead on Arrival, Medical Facility - Inpatient, Medical Facility - Outpatient or ER, Medical Facility - Status unknown, Nursing home/long term care, Other, and Place of death unknown.

From 1999 to 2020, the distribution of deaths by place of death changed significantly. The percentage of deaths at the decedent’s home increased from 8.44% in 1999 to 29.31% in 2020, while deaths in medical facilities with inpatient services decreased from 46.41% to 29.56%. Deaths in hospice facilities rose from 0.12% in 2003 to 15.15% in 2020. The percentage of deaths at medical facilities classified as dead on arrival, outpatient/ER, and unknown status decreased over time. Deaths in nursing homes/long-term care facilities also decreased from 40.24% in 1999 to 19.02% in 2020 (Fig 4).

## Discussion

Leveraging national mortality data over 22 years, our study revealed notable trends in the locations where U.S. adults die from ischemic stroke. These findings build upon and extend previous research by Cross et al., who reported increasing proportions of home and hospice deaths for individuals with cerebrovascular disease from 2003 to 2017 [13]. Our study, focusing specifically on ischemic stroke over a longer period (1999-2020), provides a more comprehensive view of these evolving patterns. Furthermore, by analyzing ischemic stroke deaths, mortality rates, and location of death collectively, we offer a nuanced picture of stroke outcomes and disparities. The observed trends in mortality rates across different demographic groups and regions provide crucial context for interpreting the shifts in location of death.

Although overall stroke mortality continued to decline from 1999 to 2010s, death rates subsequently rose across both rural and urban settings. These changes in mortality could be attributed to various factors, including shifts in case fatality rates, changes in stroke incidence, and alterations in the risk of recurrent stroke [19]. Furthermore, advances in medical therapies, surgical interventions, smoking cessation efforts, and control of key risk factors such as hypertension and hyperlipidemia likely contributed to declining mortality seen from 1999 to 2010s, as well [20–23]. The rise in deaths seen from the mid-2010s may be attributed to the increased prevalence of obesity and diabetes, advancements in diagnostic technologies, and changes in classification criteria for ICD codes [24–26].

We also observed a substantial shift away from hospitals and inpatient facilities towards private homes and hospice care settings as the location where individuals ultimately died. In summary, the data present a complex narrative of how mortality rates and place of death have evolved between 1999 and 2020, trends likely shaped by racial, urbanization, and socioeconomic influences. The marked increase in at-home deaths, alongside fluctuating mortality across racial and urban strata, underscores the need for deeper investigation into the multifaceted drivers propelling these changes.

Taken together, these dual observations of rising stroke mortality since 2010 and escalating reliance on home-based end-of-life care call for assessing the potential epidemiological, infrastructural, health policy, and access-related factors that may impact outcomes across communities nationwide. More research focused on elucidating the root causes underlying the worsening trajectories could inform targeted responses addressing disparities and preferences in stroke-related deaths.

### Contextualizing the divergent phases in mortality trends

The period from 1999-2010 showed consistent downward movement in ischemic stroke mortality, likely attributable to enhancements in preventing and treating atherothrombotic disease over recent history. Specifically, though the decrease in deaths from 2004 to 2010 is not well understood, it can be attributed to several factors. First, the decline in stroke mortality can be linked to cardiovascular risk factor control interventions in the 1970s. In more recent years, other programs targeting smoking and improved blood pressure control may have also contributed to the decrease in deaths [26].

Research points to a possible resurgence of cardiovascular mortality over the past decade driven by the increased prevalence of obesity and diabetes alongside plateaus in cholesterol and blood pressure control [23,24]. Additionally, the advancement of diagnostic technology and neuroimaging may be affecting how doctors classify cerebrovascular diseases (ICD codes I60–I69). Deaths caused by cerebrovascular disease can be particularly difficult to distinguish between, and a high error rate in classifying the cause of death for strokes has been previously reported [26].

Previous research has shown that the disparity in stroke between Black and White patients is largely due to differences in stroke incidence rather than case fatality [19]. Hence, end of greater rate of increase in mortality trends for the Black/African American group from 2018 to 2020 warrants careful consideration. One explanation for this trend could be a disproportionate increase in socioeconomic disparity, such as an increase in food deserts within the urban areas, lack of education, and proper dieting [27]. This may reflect failures around primary prevention, which compounds practical constraints in treating acute vascular events like ischemic stroke.

Our study provides corroborative evidence that ischemic stroke mortality reductions have attenuated and shifted directions amid these widening epidemics of metabolic risk factors. The broad concurrence of trend changes across rural-urban settings suggests diffuse underlying phenomena requiring increased attention. Ultimately, the disproportionate increase in the Black/African American group in stroke death compared to other racial groups shows the need for better community and systemic support for stroke detection and care [28].

While the downward stroke mortality trends continued longer for metropolitan areas with denser healthcare infrastructure, the narrowing gap between metro and rural death rates raises questions about exacerbating vulnerabilities unique to rural communities. Rural populations tend to be older, lower income, less insured, and carry higher chronic disease burdens than their urban counterpart [29]. Rural residents meeting the criteria for thrombolytic administration may still encounter barriers to accessing primary stroke centers quickly enough for treatment due to transportation logistics and care coordination challenges [10]. Such systemic constraints could disproportionately impact rural groups as therapeutic windows close. Thus, despite positive strides in lowering rural stroke mortality over the past 20 years, the closing gaps evident since 2010 highlight the importance of specifically monitoring and addressing the needs of rural communities in strategic health planning.

### Shifts towards home and hospice locations at end-of-life

Along with diverging mortality directions, the marked shift towards higher shares of ischemic stroke deaths within homes and hospice facilities underscores important societal preferences around end-of-life experiences. While over four-fifths of deaths occurred in hospitals and nursing homes at the start of the measurement period, the trend changed over two decades, with under two-thirds happening in facilities by 2020. The transition towards palliative hospice care aligns with broad recognition around enhancing patient autonomy and compassionate care support for the final days of terminal illness [30].

While the observed increase in home deaths among ischemic stroke decedents highlights important shifts in end-of-life care practices, this trend must be interpreted in context. Hospice care, for example, is increasingly being provided at home, offering specialized support to patients and families in familiar settings. Many patients transition to hospice after receiving maximal treatment in inpatient settings, and modern healthcare systems often ensure hospice access even in rural and suburban areas. Therefore, higher rates of home deaths do not universally signify a lack of access to specialized care but may also reflect evolving practices and patient preferences for home-based palliative care. The available CDC WONDER data and our analysis likely does not capture the complicated dynamics of end-of-life care. A more comprehensive dataset should be used to provide further context to these healthcare trends.

Nevertheless, disparities persist, with rural and minority populations continuing to face structural and logistical challenges in accessing end-of-life resources. Prior studies showed lower odds of hospice services for rural and Black/African American stroke patients even after adjusting for confounders like illness severity [31,32]. Financial, travel, and staffing barriers hinder rural hospice access, while cultural attitudes may inhibit open conversations around palliative options versus prolonged treatment [33–35]. Without accessible hospice care to assist home-based end-of-life transitions, rural patients risk suboptimal symptom control, caregiver burnout, and undue healthcare utilization from the impacts of terminal ischemic stroke. While reflecting a sensible preference for palliative care, the accelerating reliance on home-based end-of-life care also underscores the need to improve rural healthcare connectivity [36].

Similarly, with more limited socioeconomic resources, many Black/African American patients may face similar challenges in receiving appropriate stroke and end-of-life care [37]. Thus, while shifts towards home-based palliation conceptually align with patient preferences, compromised rural infrastructure, as well as systemic racial disparities, lead to inequities around achieving care goals.

### Interplay between race and urbanization

The data reveals that racial disparities in mortality exist across all urbanization levels but may be more pronounced in certain urban or rural contexts. Even with potentially better access to healthcare, minority groups in urban settings still face higher mortality rates than their White counterparts, indicating that access alone does not account for the disparities [37,38]. Additional patient information about insurance status, other underlying conditions, and frequency of health-related visits might be helpful in better understanding the causal relationships for disparities in mortality rates between White and minority populations.

However, it should be noted that minority groups within rural settings might face compounded disparities. For instance, the lower number of deaths among American Indians/Alaska Natives, who often reside in more rural areas, could be affected by both limited healthcare access and the smaller population size of these groups [39–40]. The relatively higher percentage of deaths while having lower total death counts overall in NonCore and Micropolitan areas for these groups may also suggest a greater impact of rural health disparities on these populations. This gives insight into how healthcare accessibility, economic limitations, and other social determinants of health impact communities disproportionately.

The interplay between race and urbanization emphasizes the need for health equity-focused interventions considering geographic and racial diversity. Addressing these disparities requires a multifaceted approach that includes bolstering rural health services, improving access to preventive and primary care in minority communities, and addressing broader socioeconomic factors that influence health outcomes. Understanding these complex relationships is crucial for developing targeted public health strategies and ensuring that healthcare systems are responsive to the needs of all segments of the population.

### Intersection of race and place of death

Our analysis of ischemic stroke deaths classified by race and location of death also revealed striking disparities. These trends are consistent with other papers’ findings that provide further context for different populations’ preferences and access to end-of-life care. For example, American Indian/Alaska Native individuals’ highest percentage of deaths in medical facilities with inpatient services possibly reflects limited access to alternative care settings in the predominantly rural areas where these populations reside [8]. On the other hand, Asian/Pacific Islander individuals had the highest rate of deaths at the decedent’s home, which may be attributed to cultural preferences for end-of-life care in home settings [11]. Black/African American individuals had the highest death rate at medical facilities with dead on arrival, suggesting potential delays in seeking timely care and challenges in accessing inpatient services [39,41]. White individuals having the lowest death rates at medical facilities with inpatient or outpatient/ER services but the highest death rates at hospice facilities and nursing homes/long-term care indicates better access, awareness, and utilization of these care options [30,31].

These findings highlight the complex interplay of racial, socioeconomic, and cultural factors in shaping end-of-life care preferences and access to services, underscoring the need for targeted interventions to address disparities and ensure equitable care for all stroke patients. Additional research should explore models enabling equitable access to specialized treatments during severe strokes alongside compassionate hospice services when curative options narrow—regardless of distance from high-volume stroke centers. With further therapeutic constraints around severe ischemic events, optimizing prevention and chronic disease management remains pivotal for ultimately stemming incidence. Targeted rural health policies and equitable, systemic public health interventions to address modifiable risk factors and access barriers around end-of-life stroke care should constitute priorities amid evolving epidemiological landscapes [42]. Comprehensive efforts focused on evidence-based primary and secondary prevention, enhancing rural healthcare capacity, and supporting patient-centered advanced care planning may help mitigate the observed worsening trajectories in ischemic stroke mortality.

### Limitations

Our analyses utilized a nationwide, population-based mortality database to elucidate evolving trends and variations in ischemic stroke deaths. However, findings should be interpreted in the context of inherent limitations with retrospective claims-based assessments and death certificate coding. Selection biases may arise from cohort identification relying on cause of death diagnoses. Rural patients may face increased barriers to obtaining autopsies needed to confirm stroke diagnoses [43]. The potential exists for stroke deaths to get misattributed to other contributing factors like myocardial infarction or atherosclerosis more broadly, though any misclassification likely remained consistent over the two decades examined.

This study utilizes the CDC WONDER database, which, while robust, is limited in its ability to account for individual-level variables such as stroke severity, specific comorbidities, and socioeconomic factors that likely influence the observed trends. Furthermore, the use of bridged-race methodology in CDC WONDER restricts the ability to separately analyze Hispanic ethnicity, an important and growing demographic group within the U.S. population. This limitation constrains the study’s ability to explore disparities specific to Hispanic individuals, who may experience unique barriers to healthcare access and outcomes. Future research should aim to incorporate datasets with finer granularity to address these gaps, enabling a more comprehensive understanding of racial and ethnic disparities in ischemic stroke mortality and care.

These limitations collectively constrain the ability to draw conclusions about the causal mechanisms underlying disparities in mortality and location of death. Future research will benefit from integrating more granular datasets that allow for detailed exploration of these confounders, improving the depth and precision of analyses.

## Conclusions

Over two decades, ischemic stroke mortality trends among U.S. adults exhibited complex trajectories, initially declining through 2010, then worsening divergently across geographical settings thereafter. In tandem, the locations where Americans died from stroke shifted substantially towards homes and hospice, especially post-2010. While disparities in mortality and location of death are well-documented in this study, further work is needed to elucidate the causal mechanisms driving these inequities. Addressing these disparities will require multifaceted strategies, including expanding telemedicine networks and mobile stroke units to bridge geographic gaps, as well as implementing culturally tailored prevention and education programs in underserved communities. These efforts should be complemented by policies aimed at reducing structural barriers to care, such as financial and transportation challenges, to improve equitable access to end-of-life care.

## Supporting information

S1 File CDC WONDER data query parameters. (DOCX)
