# Correction: Trends and disparities in ischemic stroke mortality and location of death in the United States: A comprehensive analysis from 1999–2020

**DOI:** 10.1371/journal.pone.0341830

**Published:** 2026-01-27

**Authors:** Jason K. Lim, Jenlu Pagnotta, Richard Lee, Do H. Lim, Jeffrey M. Breton, Zachary A. Abecassis, Raymond M. Meyer, Jeffrey C. Mai, Michael R. Levitt

The Data Availability statement for this article is incorrect. The correct Data Availability statement is as follows: The data underlying the results from this study are publicly available from the CDC WONDER database (https://wonder.cdc.gov/ucd-icd10.html). Researchers can access the data by utilizing the search parameters provided in the Supporting Information (S1 File).

In the Place of death by race subsection of the Results, there is an error in the fourth sentence of the first paragraph. The correct sentence is: White individuals had the lowest death rate at medical facilities with inpatient or outpatient/ER services but the highest death rate at hospice facilities (8.58%) and nursing home/long-term care (27.87%).
