# Supplementary material for: Trends and disparities in ischemic stroke mortality and location of death in the United States: A comprehensive analysis from 1999–2020
Source: PLoS One. 2025 Apr 9;20(4):e0319867. doi: 10.1371/journal.pone.0319867 (PMC11981169; doi:10.1371/journal.pone.0319867)
Supplement: S1 File — (DOCX) [file pone.0319867.s001.docx]

**CDC WONDER data query parameters:**

**Dataset:** Underlying Cause of Death, 1999-2020

- **Organize table layout:**

‘Group Results By’: Race, Place of Death, Year, Ten-Year Age Groups, 2013 Urbanization.

‘Measures’: Age Adjusted Rate

‘Calculate Rates Per’: 100,000

- **Cause of death:**

ICD-10 Codes: I63.0 (Cerebral infarction due to thrombosis of precerebral arteries); I63.1 (Cerebral infarction due to embolism of precerebral arteries); I63.2 (Cerebral infarction due to unspecified occlusion or stenosis of precerebral arteries); I63.3 (Cerebral infarction due to thrombosis of cerebral arteries); I63.4 (Cerebral infarction due to embolism of cerebral arteries); I63.5 (Cerebral infarction due to unspecified occlusion or stenosis of cerebral arteries); I63.6 (Cerebral infarction due to cerebral venous thrombosis, nonpyogenic); I63.8 (Other cerebral infarction); I63.9 (Cerebral infarction, unspecified)
